# Key Gaps in the Knowledge of the Porcine Respiratory Reproductive Syndrome Virus (PRRSV)

**DOI:** 10.3389/fvets.2019.00038

**Published:** 2019-02-20

**Authors:** Sergio Montaner-Tarbes, Hernando A. del Portillo, María Montoya, Lorenzo Fraile

**Affiliations:** ^1^Innovex Therapeutics S.L, Badalona, Spain; ^2^Departamento de Ciencia Animal, Escuela Técnica Superior de Ingenieria Agraria (ETSEA), Universidad de Lleida, Lleida, Spain; ^3^Germans Trias i Pujol Health Science Research Institute, Badalona, Spain; ^4^ISGlobal, Hospital Clínic—Universitat de Barcelona, Barcelona, Spain; ^5^Institució Catalana de Recerca i Estudis Avançats, Barcelona, Spain; ^6^Centro de Investigaciones Biológicas, Consejo Superior de Investigaciones Cientificas, Madrid, Spain

**Keywords:** porcine reproductive and respiratory syndrome virus, PRRSV, virus biology, immunology, vaccinology, extracellular vesicles

## Abstract

The porcine reproductive and respiratory syndrome virus (PRRSV) is one of the most important swine diseases in the world. It is causing an enormous economic burden due to reproductive failure in sows and a complex respiratory syndrome in pigs of all ages, with mortality varying from 2 to 100% in the most extreme cases of emergent highly pathogenic strains. PRRSV displays complex interactions with the immune system and a high mutation rate, making the development, and implementation of control strategies a major challenge. In this review, the biology of the virus will be addressed focusing on newly discovered functions of non-structural proteins and novel dissemination mechanisms. Secondly, the role of different cell types and viral proteins will be reviewed in natural and vaccine-induced immune response together with the role of different immune evasion mechanisms focusing on those gaps of knowledge that are critical to generate more efficacious vaccines. Finally, novel strategies for antigen discovery and vaccine development will be discussed, in particular the use of exosomes (extracellular vesicles of endocytic origin). As nanocarriers of lipids, proteins and nucleic acids, exosomes have potential effects on cell activation, modulation of immune responses and antigen presentation. Thus, representing a novel vaccination approach against this devastating disease.

## Economic Impact

PRRSV is responsible for respiratory disease in weaned and growing pigs, as well as reproductive failures in sows. It is considered one of the most important swine diseases worldwide, with an economic impact estimated at $664 million in losses every year to U.S. producers, representing an increase of 18.5% in the last 8 years (1, 2). In Europe, the situation is similar and economic disease models have been carried out to determine the economic burden in the best and worst case scenario combining reproductive failure and respiratory disease, estimating annual losses from a median of €75,724, if the farm was slightly affected during nursing and fattening, to a median of €650,090 if a farm of 1,000 sows is severely affected in all productive phases (3). Nevertheless, there is scarce of information about the economic impact of this disease as a consequence of multiple factors (vaccination, treatment, respiratory symptoms, reproductive failure, and other PRRSV-related diseases) making a difficult task to quantify exactly this parameter under field conditions. Thus, the exact economic impact of PRRSV remains a key gap in the knowledge for this disease.

## Biology of PRRSV

**Figure 1:**
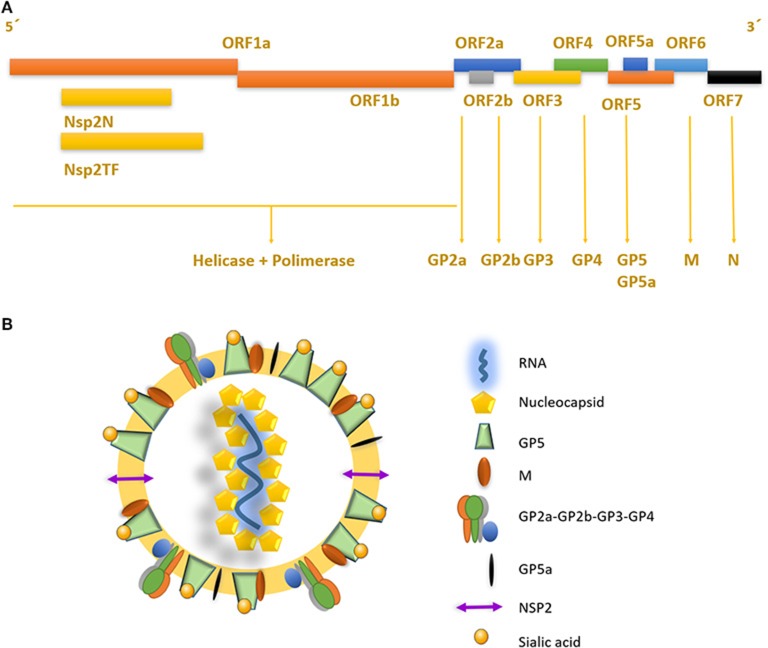
Genome structure and mature viral particle of PRRSV virus. **(A)** Non-structural proteins are located in the 5′ end of the genome, codifying for two different polyproteins pp1a and pp1ab that are cleaved into at least 14 nsps (nsp1 to nsp12 and nsp1α and nsp1β, and nsp7α, and nsp7β). Structural proteins located near the 3′ end, are associated to the viral envelope and RNA packaging. **(B)** PRRSV mature viral particle, composed of a lipid bilayer envelop with viral receptor glycoproteins involved on infection and cell internalization. Single stranded positive RNA is associated with nucleocapsid protein in the internal layer of the virus.

The porcine reproductive and respiratory syndrome virus (PRRSV) was first isolated in the early 1990s in Europe and North America (4, 5). It is an enveloped single-stranded positive-sense RNA virus of the family *Arteriviridae*, Genus *Porarterivirus* according to the International Committee of Taxonomy of Viruses (6). Presently, there are four distinct species included in this Genus (*Porarterivirus)*, PRRSV-1 and PRRSV-2 (with 30–45% variation in nucleotide sequences), along with other two viruses that do not affect pigs (Lactate dehydrogenase-elevating virus and Rat Arterivirus 1) (7). The genome size of PRRSV is about 15 kb with 10 open reading frames (ORFs), with replicase genes located at the 5′-end followed by the genes encoding structural proteins toward the 3′-end (8). The majority of the genome (~60–70%) encodes non-structural proteins involved in replication (ORF1a and ORF1ab), whereas ORFs 2–7 encodes structural proteins (N, M, GP2-GP5, E) (Figures 1A,B) (9). Using ORF5 in molecular epidemiological studies, an enormous genetic variability has been described (10). Yet, data on whole genome sequencing is scarce and constitute another important gap in the knowledge of this virus and its evolution (Box 1).

Box 1 **Gaps in knowledge in PRRSV.**

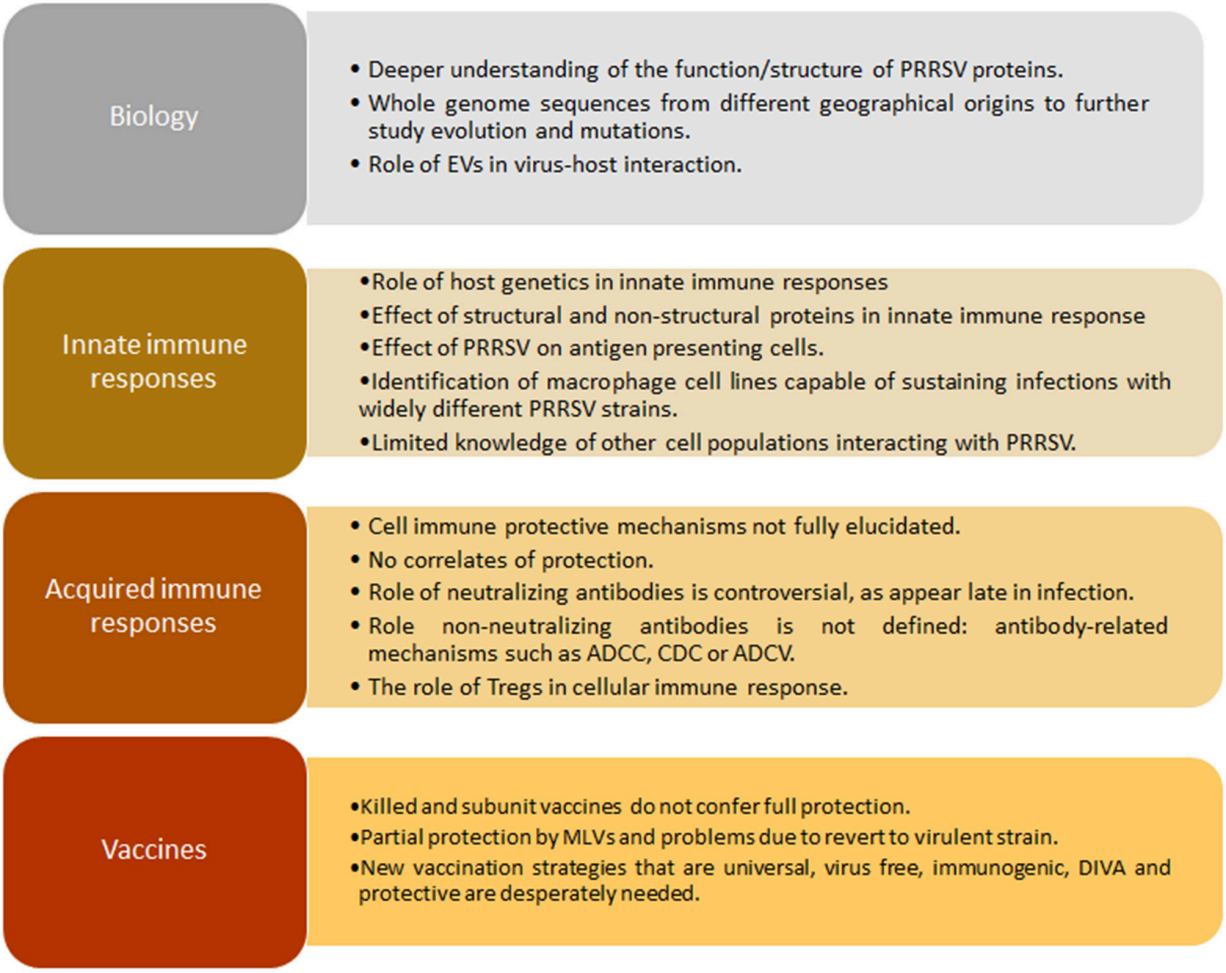

PRRSV replicase genes consist of two ORFs, ORF1a and ORF1b, which occupy the 5′ proximal three-quarters of the genome (Figure 1A). Both are expressed from the viral genome, with expression of ORF1b depending on a conserved ribosomal frameshifting mechanism. Subsequently, extensive proteolytic processing of the resulting pp1a and pp1ab polyproteins yields at least 14 functional non-structural proteins (nsps), specifically nsp1 to nsp12, with both the nsp1 and nsp7 parts being subject to internal cleavage (giving origin to nsp1α and nsp1β, and nsp7α, and nsp7β, respectively), most of which assemble into a membrane-associated replication and transcription complex (11). Recently, a programmed ribosomal frameshift encoding an alternative ORF that generates two extra proteins, nsp2TF and nsp2N, was discovered in PRRSV and other *Arteriviruses* (12, 13). These nsps, described for PRRSV, have proven to be necessary and sufficient for the induction of membrane modifications resembling those found in infected cells (14). Most importantly, all positive RNA viruses seem to induce one of two basic morphotypes of membrane modifications: invaginations or double-membrane vesicles.

**Figure 2:**
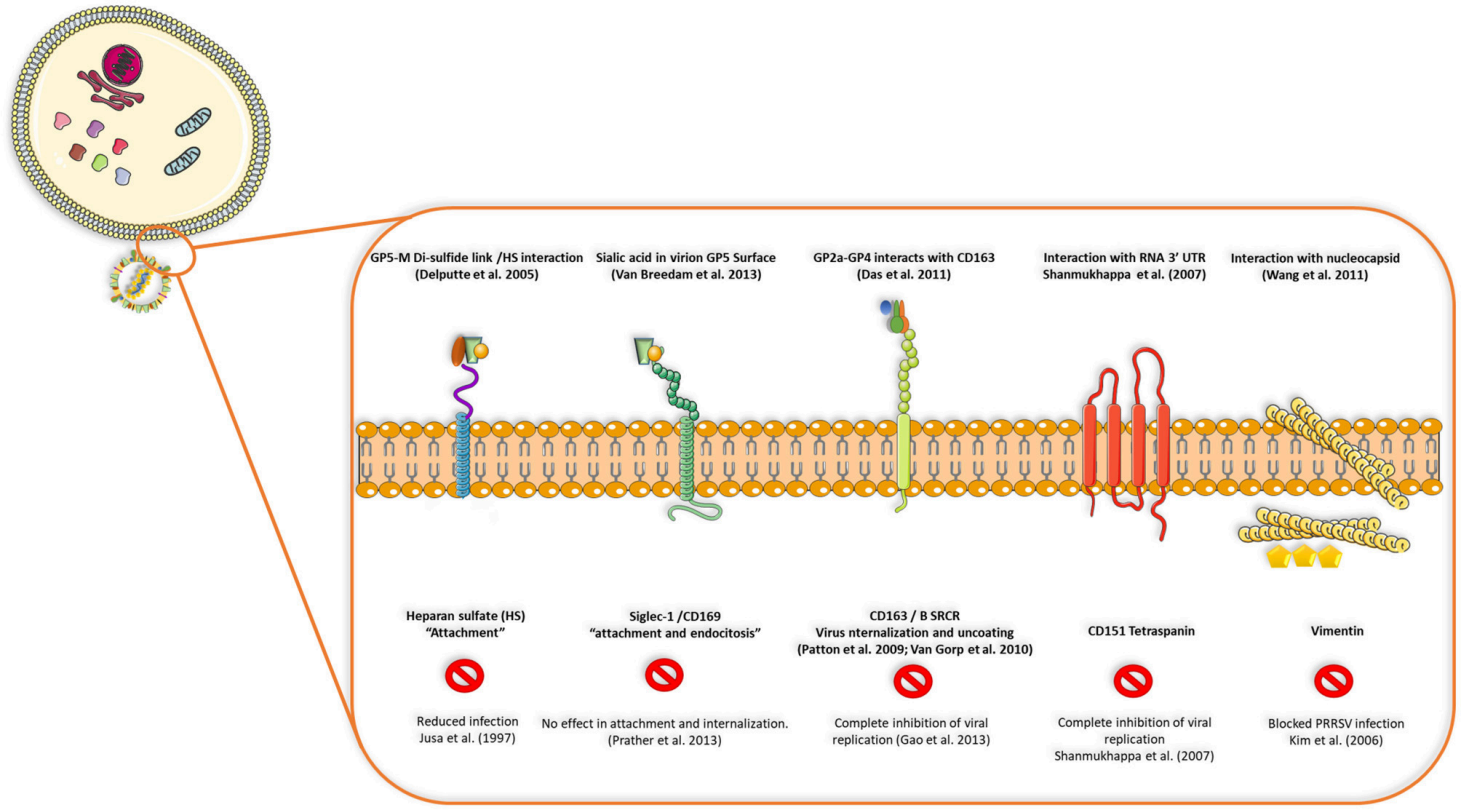
Interactions between viral proteins and cell receptors for virus attachment, entry, uncoating and release of genetic ssRNA to cell cytoplasm. Blocking CD163, CD151 tetraspanin or vimentin seems to inhibit viral replication or infection in the host cell, but reduced replication or no effect is seen when receptors such as heparan-sulfate or siglec-1 are blocked, demonstrating that some viral proteins and cell receptors are indispensable in terms of production of infectious viral progeny and dissemination in the host.

PRRSV also has a set of 8 structural proteins, including a small non-glycosylated protein and a set of glycosylated ones: GP2a-b, GP3, GP4, GP5, and GP5a, M and N proteins (15). However, nsp2, traditionally classified as a non-structural protein, has been found to be incorporated in multiple isoforms within the viral envelope (Ovarian tumor domain protease region, hypervariable region and C-terminal region) (16), giving new insights into the structure of this virus (Figure 1B). First, the nucleocapsid protein (N), as one of the most important parts of the mature viral particle, has been deeply characterized on PRRSV, finding important features shared in most non-segmented RNA viruses. The N protein consists of 123 amino acids for genotype 2 and 128 amino acids for genotype 1. The viral envelope glycoproteins (GP2 to GP5) are the first interactors with host cell receptors to initiate infection and are exposed to the immune system when viral particles are in blood and lymphoid tissue circulation (Figure 2). There is also another protein that contribute to virion structure, M protein, that is required during viral entry to interact with heparan sulfate cell receptor on macrophages. Later, GP5 is thought to bind to sialoadhesin and virus internalization and uncoating is triggered by a formation of a viral heterotrimer (GP2a, GP3, and GP4) with scavenger receptor CD163 (Figure 2) (17, 18). GP5 is the most abundant glycoprotein. First, it interacts with two cell entry mediators, heparan sulfate glycosaminoglycans and sialoadhesin/CD169 (17, 18) to favor viral entry and then possibly with the N protein and its MHC-like domain to carry N-Viral RNA complex to the budding site (Figure 2). GP2, GP3, and GP4 are protected with glycan shields, like most PRRSV membrane proteins, to avoid antibody recognition and neutralization. GP2 has two glycosylation sites, GP3 have seven and GP4 have four, three of which are directly related to virus survival, causing lethal damage in virus production when more than two of these sites are mutated (19) (Figure 2).

## Virus Replication and Entry Mechanisms in Host Cells

Viral replication starts by interaction of viral glycoproteins with different cellular receptors (Figure 2) (17). CD163 and CD169 play a main role during infection, uncoating of the viral particle, activation of clathrin-mediated endocytosis and release of viral genome in the cytoplasm (20). CD163 has been defined as the main receptor for viral infection by evaluating the effect of PRRSV on CD163 knockout pigs, where there is complete resistance to infection (21). Cysteine-rich domain 5 in this receptor seems to be necessary to establish interactions with PRRSV-1 species, since its deletion by CRISPR/Cas9 system (exon 7 of the gene encoding this region) implies protection for a large panel of these viruses demonstrated by *in vitro* challenge of edited-pig macrophages and *in vivo* experiments with ΔSRCR5 animals (22–24). More important, edited pigs show no side effects when kept under standard husbandry conditions and CD163 seems to maintain its biological function (hemoglobin-haptoglobin scavenger) regardless the lacking cysteine-rich 5 domain, nevertheless, other unknown functions could be impaired by this modification. In conclusion, gene-edited pigs lacking SRCR5 region of CD163 could be an important asset to confront PRRSV epidemics with the final goal of eradication.

CD169 seems to be related only to co-interactions with sialic acid in the virion surface, however, knockout pigs for either exon 1, 2, or 3 of CD169 were not protected from infection and viral load as well as antibody responses were similar to heterozygous (CD169^+/−^) or wild type pigs (CD169^+/+^) (25). The former experiments suggested that other unknown mechanisms could be involved in PRRSV infection such as other receptors, new unknown susceptible cell types different from macrophages or possible leaking of CD169 expression in the knockout model.

Other molecules are also involved in viral entry, such as CD151 (26) and vimentin (27); blocking of any of these four molecules (CD163, CD169, CD151, and vimentin) had an effect on viral infection, either on internalization or complete inhibition of viral replication (17). After cell entry, PRRSV causes a series of intracellular modifications to complete its replication cycle, which includes rearrangements of intracellular membrane organelles to generate the replication complex. These include the formation of perinuclear double membrane vesicles apparently derived from endoplasmic reticulum, synthesis of genomic RNA (gRNA), transcription of segmented RNA (sgRNA) and expression of viral proteins (20, 28). At late stages of replication, the mature virions accumulate in the intracellular membrane compartments and they are then released into the extracellular space through exocytosis (29).

A non-classical spread pathway has been detected in several viruses including PRRSV where virus dissemination is mediated by cell to cell nanotubules (30). It was reported that almost all PRRSV proteins interact with myosin and actin (especially F-actin and Myosin IIA) where nanotubules connected cells allowing the movement of structural proteins and RNA, infecting naïve cells in a non-classical way even in the presence of neutralizing antibodies in the cell media. In addition, this non-classical pathway demonstrated that PRRSV cell entry receptors were not necessary to establish infection, as non-permissive cells became infected when were contacted by infected cells via nanotubes. This spreading strategy has been proposed as a mechanism to facilitate infection either by surfing of viral particles between adjacent cell membranes or as a receptor-independent mechanism for infection (31); Importantly, has been reported for other viruses such as HIV-1 where nanotube number on macrophages increases after infection (32) and Herpesvirus transmission between bovine fibroblasts (33). Interestingly, although several viral proteins were detected in nanotubules (nsp1β, nsp2, nsp2TF, nsp4, nsp7, and nsp8, GP5 and N), GP4 was detected in only a few nanotubes. In particular, the role of GP4 in this non-classical spread pathway is not fully understood and it will be interesting to further evaluate GP4 interaction with other cellular components to elucidate the reason why GP4 is not transported to new recipient naïve cells. Altogether these data indicate that PRRSV has evolved different pathways to spread even though, *in vivo*, the virus shows narrow cell tropism for monocytes and macrophages (34, 35) (Box 1).

## Immunology of PRRSV and Mechanisms Involved in Immune Evasion

### Innate Immune Response

The innate immune response is the first system any given pathogen encounters, specially to prevent viral replication and invasion into mucosal tissues (respiratory tract in the case of PRRSV) and, importantly, to initiate the strong adaptive immune response to fight against intracellular infectious agents (7). Type I interferons (IFN α/β) comprise one of the most potent mechanisms against invading viruses in the first stages of infection, triggering an array of IFN-stimulated genes (ISG) (36). Generally speaking, all nucleated cells have the ability to produce IFN α/β, but plasmacytoid DC (pDC) are the most potent producers of this family of cytokines (37). PRRSV has evolved a set of mechanisms for suppressing IFN α/β *in vivo*, maintaining low expression levels of this cytokines on infected pigs (38) during almost all time-course of infection shortly after transient elevation in the lungs (39). Suppression of IFN α/β also takes place *in vitro* in PRRSV infected MARC-145 and porcine alveolar macrophages (38, 40, 41). Further studies have shown that IFN type I suppression is a major strategy of PRRSV to modulate host antiviral defense. In fact, several viral proteins have been identified as IFN antagonists (nsp1α, nsp1β, nsp2, nsp4, nsp11, and N) (7, 42–44). As an example for N protein, upon dsRNA stimulation, IFN-β production was shown to decrease proportionally with increasing levels of N expression and additionally it was found to downregulate IFN-dependent gene production by dsRNA interfering with dsRNA-induced phosphorylation and nuclear translocation of IRF3 (45).

Among PRRSV non-structural proteins with type I IFN modulation capacity, nsp1 has been considered as the strongest antagonist of IFN-β production by acting on interferon regulatory factor 3 (IRF3) phosphorylation and nuclear translocation. Almost all nsps, excepting nsp1, have been related to the perinuclear region, associated with intracellular membranes, supposedly derived from the endoplasmic reticulum (ER), which are modified into vesicular double-membrane structures with which the viral replication and transcription complex (RTC) is thought to be associated with (14, 46, 47). Nsp1 translocates to the nucleus during the first hours of infection, where it is capable of inhibiting IRF3 association with CREB-binding protein (CBP), promoting CBP degradation by a proteasome-dependent mechanism, without which the transcription enhanceosome may not assemble the transcription machinery for the interferon expression (15, 46). Recently, post-transcription protein expression of IFN β was shown to be regulated by PRRSV by means of upregulating cellular miRNA in porcine alveolar macrophages (48)

Nsp2 is the largest (mature) PRRSV protein and contains at least four distinct domains: The N-terminal CP/OTU domain, a central hypervariable region, a putative transmembrane domain, and a C-terminal region of unknown function that is rich in conserved cysteine residues. This protein is unique in the context of PRRSV due to its genetic heterogeneity, its participation in diverse roles supporting the viral replication cycle, and its packaging within the PRRSV virion (16, 49). Previous studies suggest that nsp2 has different roles related to immune evasion mechanisms. It has been determined that nsp2 OTU domain (thiol-dependent deubiquitinating domain) inhibits the nuclear factor kappa-light-chain-enhancer of activated B cells (NF-kB) by interfering with the polyubiquitination process of IkBα (nuclear factor of kappa light polypeptide gene enhancer in B-cells inhibitor) and, subsequently, preventing the degradation of the IkBα protein (50). Moreover, viable deletion mutants in nsp2, when infecting cells, caused a downregulation of cytokines (IL-1β and TNF-α) mRNA expression, in comparison with that of parental virus, suggesting that certain regions of nsp2 might contribute to the induction of a virus-specific host immune response and that deletion of such a region could produce a more virulent virus (51).

There are several isoforms of nsp2, sharing a consistent core set between viral strains, which are integrated into mature virion at the final stage of replication (Figure 1B), although some of them could be strain-specific. Inclusion of nsp2 within the PRRSV virion suggests that it may function in previously unknown roles related to extracellular function, entry, or immediate-early viral replication events (16). Truncated forms of nsp2 have also been identified, named nsp2TF and nsp2N, with apparent roles in modulation of immune evasion. When deletion mutants for those forms were used to infect cells, there was a significant change in gene expression, a strong activation of those involved in cytokine-cytokine receptor interaction, TNF signaling, toll-like receptor signaling, NOD-like receptor signaling, NF-κB signaling, RIG-I-like receptor signaling, chemokine signaling, JAK-STAT signaling, cytosolic DNA-sensing, and NK cell mediated cytotoxicity (13), suggesting that an active role (direct or indirect) is played by these truncated forms in modulating host cells innate immune response, making PRRSV infectious cycle more complicated than it was initially thought.

Nsp11, is a *Nidovirus* conserved endoribonuclease with an uridylate-specific endonuclease (NendoU). It has been demonstrated *in vitro* that overexpression of nsp11 enhanced viral titter (52). Moreover, nsp11 antagonizes type I IFN, specifically IFNβ production, activated by the retinoic acid inducible gene 1 like receptor, showing substrate specificity toward Mitochondrial Antiviral Signaling proteins (MAVS) and RIG-I (transcripts and proteins), and demonstrating that this activity was associated to the endoribonuclease activity of this protein in which transfection mutant viruses were unable to degrade MAVS mRNA and impair IFNβ production (53). Another mechanism whereby this protein limits antiviral response is related to inflammasome and synthesis of IL-1β, due to its important role in both the innate and adaptive immune response and in pathological mechanisms. It has been shown that PRRSV could activate NLRP3 inflammasome in early stages of infection but induce host's immunosuppression later as measured by determining the levels of pro-IL-1β and procaspase-1 mRNA and the mature IL-1β protein in porcine alveolar macrophages (PAM) (54). It is not surprising that nsp11 also interacts with the RNA-silencing complex (RISC), as it has been demonstrated *in vitro* in a MARC-145 cell line that this protein and nsp1α are responsible for inhibiting RISC and downregulating argonaute-2 protein expression increasing viral titter significantly, which demonstrates a direct relationship between this silencing complex and viral replication at least *in vitro* (55).

Other non-structural proteins have been studied but there is an important gap on information about *in vivo* and *in vitro* functions and interaction in signaling pathways. Additionally, the enormous variation among strains makes it difficult to characterize all protein variants and interactions with cell systems (macrophages, Dendritic cells “DCs,” monocytes and others) (Box 1).

Recently, a body of evidence associates host genetics with different outcomes following PRRSV infection in the respiratory and reproductive form of the disease (56–60). Although pathways and mechanisms involved in specific disease-resistance traits have not yet been fully characterized, it is clear that the genetic variation in disease resilience is polygenic, regulating aspects of both innate resistance and acquired immunity (56). In connection with innate response, the average daily gain (ADG) after PRRSV infection was associated with a single genomic region in chromosome 4 (SSC4) which is best represented by the SNP tag marker WUR, located in the 3′ non-coding region of the interferon-inducible guanylate-binding protein 1 (GBP1) gene (61). The pig genetic resistance to PRRSV infection has been historically overlooked in PRRSV research probably generating a confounding factor in immune response studies. A key gap in the knowledge of PRRSV is linked the pig genetic variability after PRRSV infection with the enormous variability of the virus itself (Box 1).

In pigs, PRRSV replicates in cells belonging to the innate immune system. PAMs are the primary cells to be infected in the lungs as well as other cells of the monocyte/macrophage lineage, which later could disseminate the virus to other tissues or support replication to release viral particles into the bloodstream (17) (Figure 2). Moreover, PRRSV is thought to be able to infect professional antigen presenting cells such as DCs and monocyte derived dendritic cells, (MoDC) impairing their normal antigen presentation ability by inducing apoptosis, down-regulating the expression of IFN-α, MHC class I, MHC class II, CD11b/c and CD14, upregulating the expression of IL-10 and inducing minimal Th1 cytokine secretion (62–65). Nevertheless, new evidence suggest by *in vivo* and *in vitro* experiments that specifically lung cDC1, cDC2, and MoDCs are not infected by PRRSV-1 viruses from subtypes 1 and 3 and one possible explanation is the lower expression of CD163 and CD169 in those 3 DC subtypes, associating previous results of infection in DCs to culture conditions of monocytes *in vitro* that could cause a sensibilization to infection by certain strains as Lena (66). In addition, these findings were also tested in tonsil cDC and tracheal cDC1 and cDC2 observing that those cell populations are not infected by PRRSV virus (67, 68).

Moreover, a new type of PAM has been characterized and named porcine intravascular macrophages (PIM) due to its association to endothelial lung capillaries and not to the alveoli, presenting strong capacity to phagocytised blood-related particles (69). Importantly, when infected PIM cells gave similar results of viral load to those derived from infected PAM, but significantly upregulates of TNFα and non-significantly IL-6 and IL-8 expression after infection when compared to normal alveolar macrophages, indicating that these cells have an important pro-inflammatory role during PRRSV infection in the lungs (69). New interactions between cells and the virus need to be further explored to unravel possible immunological features that leads to correlates of protection.

Recently, it has been shown that a domain within Nsp1α is able to stimulate the secretion of CD83, which in turn inhibits MoDC function *in vitro*, impairing the ability of MoDC to stimulate T cell proliferation (70). Production of IFN α/β and the mechanisms for cell activation by pDC are severely suppressed during PRRSV infection, although these cells are not permissive to PRRSV infection (71, 72). However, this phenomenon is strain dependent, as other PRRSV strains are able to stimulate pDC for IFN α/β production in large quantities (73). Again, there is an enormous variability between PRRSV strains in relation with their effect on antigen presenting cells which prevent scientists from finding common mechanisms. It might be of interest to link this key gap of knowledge for PRRSV with host genetics (Box 1). Moreover, in PRRSV-infected cells, N is abundantly expressed benefiting from the discontinuous transcription mechanism (74). This protein is also distributed in the nucleus, induced by two nuclear localization signals called cryptic NLS or NLS-1 and functional NLS or NLS-2 (positions 10–13 and 41–47, respectively) (75). The effect of N protein has been examined in PAMs and MoDCs using transfection, finding a significant upregulation of IL-10 gene expression.

Natural killer (NK) cells constitute another powerful arm of the innate immune system against PRRSV, particularly when considering the high percentage of circulating NK cells in pigs (76). The cytotoxic function of NK cells is reduced in PRRSV infected pigs from day 2 after infection up to 3–4 weeks (38, 77, 78). Initial studies using *in vitro* systems demonstrated that the stimulation of porcine NK cells with proinflammatory cytokines (IL-2 and IL-15) was capable of activating NK cells and inducing them to express high levels of IFN-γ and perforins to cause lysis of infected cells, but a different scenario appears if cells are evaluated post-infection, indicating that a virus such as PRRSV is capable of impairing NK cell cytotoxicity (79). *In vitro*, the NK cytotoxicity against PRRSV-infected PAMs was decreased and degranulation of NK cells inhibited (80). *In vivo*, the immune response is the same as that observed *in vitro*, with some studies reporting that approximately half of viremic pigs had a reduction >50% in NK cell-mediated cytotoxicity and enhanced secretion of IL-4, IL-12, and IL-10 and reduced frequency of cytotoxic T-cells (CD4^−^CD8^+^ T) and double positive T cells (CD4^+^CD8^+^ T) and upregulated frequency regulatory T- cells (Tregs) (81).

### Acquired Immune Responses

Innate immune responses against PRRSV are obstructed by different mechanisms as are adaptive responses. The modest and delayed B cell mediated neutralizing antibody response is one of the main characteristics associated to PRRSV acquired immune responses. Even though PRRSV specific antibodies appear early at 7–9 days post-infection, the efficacy of those antibodies remains unclear. Neutralizing antibodies take longer, appearing nearly 1 month after infection (34). However, passive transfer of these neutralizing antibodies conferred almost full protection in a PRRSV reproductive model (95% of offspring alive after challenging pregnant sows with high neutralizing antibody titter). Nevertheless, in another experiment using the reproductive model, when the presence of PRRSV was examined after the transfer of neutralizing antibodies, lungs, tonsils, buffy coat cells, and peripheral lymph nodes contained replicating PRRSV similar to infected controls, although pigs were apparently protected against infection. In summary, passive transfer of high neutralizing antibody titter conferred protection to gilts and offspring (not detectable viremia), but did not eliminate the presence of viral particles in peripheral tissues nor transmission to animals they were in contact with (82–84). Curiously, the role of neutralizing antibodies in the protection against the respiratory form of the disease is a key gap of knowledge for PRRSV. This point is critical to define precisely targets for improved vaccines based on the humoral immune response against this virus (Box 1).

N protein is involved in several mechanisms for immune evasion and is also one of the most immunogenic structural proteins (75). Antibodies against N appear early during acute infection, together with those against M and GP5 proteins, but are non-neutralizing and could be involved in antibody dependent enhancement (85, 86).

There are other “antibody-related mechanisms” that do not necessarily involve neutralizing activity. Antibody-dependent cell-mediated cytotoxicity (ADCC), antibody-dependent complement-mediated cytotoxicity (CDC) and antibody-dependent complement mediated virolysis (ADCV) have been examined in the context of PRRSV, although none of these mechanisms were evident during infection or have not been deeply investigated on *in vitro* and *in vivo* models of this virus (87). It is important to note that neutralizing antibodies appear late in PRRSV infection and other immune mechanisms (cellular or antibody mediated immune response) might be acting to suppress viral replication in blood, causing the virus to be isolated in lymphoid tissues and maintaining suboptimal replication that will finally end in viral clearance. For type PRRSV-2 it has been demonstrated that immunization of pigs with ectodomain peptides from GP5/M complex did not induce neutralizing antibodies (88) although those ectodomain-specific antibodies generated were capable of binding virus.

An important feature that makes difficult to validate the location of neutralizing epitopes is the number of glycosylations in or around it. For PRRSV-1 strains, up to 3 glycosylations may be found in, or flanking the GP5 neutralizing epitope that is located between amino acids 37–45 (89), whereas for PRRSV-2 strains there are four potential glycosylation sites (90). When tested, PRRSV with mutations in GP5 glycosylation sites (either at N44 or in the hypervariable region, upstream the neutralizing epitope) enhanced immunogenicity with increased concentration of antibodies directed to this epitope 5–10 fold higher compared with those induced by the wild type strains (89). Same results were obtained when administering another deglycosylation mutant (double deglycosylation in the putative glycosylation moieties on GP5) twice, which conferred better protection against homologous challenge (91). In addition, when this protein is expressed early during infection, it stimulates production of early neutralizing antibodies and IFN-β, two main antiviral mechanisms, demonstrating its role in induction of self-protection mechanisms from the host (92). Available data about neutralizing antibodies induced by this protein are controversial, which may be due to the high variation among PRRSV strains (93) and, as previously commented, the host genetics. ORF5 is also complemented by a small frameshift of the subgenomic mRNA called ORF5a, encoding a type I membrane protein consisting primarily of alpha helix with a membrane-spanning domain (called GP5a) that is incorporated into virions as a very minor component, playing a role in viral replication, as mutation in the initiation codon or premature termination related to expression for this protein leads to non-efficient viral replication and lower titter (94, 95). This protein is capable of eliciting specific antibody immune response in natural infections and after immunizations, although those are not neutralizing neither protective in a challenge trial after infection, making difficult to define the role of this particular small protein in the whole immune response and viral clearance of PRRSV infection (96). In summary, the role of humoral immunity remains elusive in PRRSV infection (neutralizing and non-neutralizing antibodies) and a better characterization will be required to overcome this relevant gap of knowledge (Box 1).

Treg typically increase in number in chronic viral diseases to prevent a persistent inflammatory response and pathological damage associated to viral infections. Conversely, Tregs are described as key contributors in modulating the host immune response to viral infection. This cell population is an important component in regulating the magnitude of the immune response to infection (in viruses such as HIV and HCV), thus preventing excessive inflammation and tissue damage. However, they can also be inappropriately induced by viruses to switch the balance of the immune response in favor of maintaining viral replication (97). In PRRSV, the role of Tregs remains unclear and appears to be a consequence of IL-10 induction of some strains as early as 2 days post infection (81). In some experiments, *in vitro* infected DCs with PRRSV-1 exhibited an unbalanced ability to stimulate T cell immune responses in a strain-dependent manner, but no Tregs were detected, at least *in vitro*, as measured by expression of CD25 and FoxP3 markers (98). When using PRRSV-2 strains, the case seems to be different, as the virus was capable of stimulating IL-10 production with concomitant generation of Tregs (99) which was associated to nucleocapsid protein expression in the *in vitro* system. This group also suggested that IL-10 production and Treg could be related to impaired gamma interferon (IFN-γ) production and altered development of protective T-cell response by inhibiting T-cell proliferation as seen in the early stage of infection with viruses such as HCV. Vaccine strains currently in use in the United States do not provide adequate heterologous protection, one possible explanation could lay on their inability to induce an adequate IFN-γ response due to their ability to stimulate Tregs, at least *in vitro* (100). Structural conformation, but not nuclear localization, of the expressed N protein was suggested as essential for the ability to induce IL-10 that, in consequence, causes induction of Tregs as measured by markers CD4+CD25+Foxp3+ (99). It should be noted that when the role of the nuclear localization signal was evaluated using deletion mutants, results suggested that NLS-2 was not essential for virus survival, although pigs developed a significantly shorter duration of viremia and higher neutralizing antibodies than those of wild-type PRRSV-infected pigs (101). The role of Tregs cells in the immune response against PRRSV is a key gap of knowledge in order to develop more efficacious PRRSV vaccines (Box 1).

Moreover, reports have highlighted the impact of PRRSV infection on thymic cellularity mainly as a loss of CD4^+^/CD8^+^ cells in the thymus of PRRSV-infected pigs. Acute lymphopenia, thymic atrophy, and lymphadenopathy associated with the presence of PRRSV antigen in the thymus are some of the mechanisms whereby PRRSV suppresses the immune response. In addition, presence of PRRSV antigens in the thymus could also induce tolerance and presents a mechanism that could explain the presence of Tregs during PRRSV infection (93). Nevertheless, the picture is not complete and basic knowledge about the effect of PRRSV on cell development in the thymus would be of great interest to understand the effect of this viruses in the host.

PRRSV immunology thus remains an unsolved puzzle due to complex interactions between different viral strains and the host. Similar immune responses could be the key feature of this virus, such as persistence viremia, a strong inhibition of innate cytokines (IFN-α/β, TNF-α, IL-1β, IFN-γ), dysregulation of NK cell function (cytotoxicity and degranulation), rapid induction of non-neutralizing antibodies, delayed appearance of neutralizing antibody, late and low CD8+ T-cell response, and induction of regulatory T cells (Tregs) (102). As a whole, neutralizing antibodies and PRRSV-specific IFN-γ secreting cells do not fully depict the immune effector functions related to protective immunity, as the viral targets related to them are unknown. As a consequence, correlates of protection remain elusive for this infection due to the laborious work *in vitro* and *in vivo* and the enormous genetic diversity that causes confusion and makes it difficult to predict how immune responses against one isolate or strain could be applied to another in a cross-protective immune prediction model (103, 104). Without any doubt, the most important gap of knowledge for PRRSV is the lack of correlates of protection that makes extremely difficult to have robust models to check vaccines efficacy against this disease (Box 1).

### Vaccination Strategies in PRRSV. Classical and Novel Vaccines

Since the beginning of PRRSV outbreaks in Europe and the USA, the development of efficacious PRRSV vaccines has been a challenge. Classical approaches are not working properly for several reasons: viral mutation can lead to more pathogenic strains, there is a lack of knowledge on how the porcine immune system interacts with all PRRSV proteins, and most importantly, there is no robust parameter (surrogate marker) that can be unequivocally linked with viral clearance. Thus, there is no relationship between complete homologous or heterologous protection and classic immunological parameters such as an increase/decrease in particular cell population (105), IFN-γ production, neutralizing antibodies (106), non-neutralizing antibodies and clinical outcome (107). In addition, highly divergent strains make it more difficult to develop a universal vaccine for this virus (28).

Several different vaccines against PRRSV have reached the market and have been reviewed recently (108). Most of these vaccines rely upon modified live virus (Porcilis PRRS from Merck, Ingelvac PRRSFLEX EU from Boehringer Ingelheim, Amervac-PRRS from Hypra, Pyrsvac-183 from Syva) against PRRSV-1, as well as some to control PRRSV-2 (Fostera PRRS from Zoetis, Ingelvac PRRS MLV/Ingelvac PRRSATP from Boehringer Ingelheim). There is also evidence that most MLV vaccines of both PRRSV-1 and PRRSV-2 species elicit specific humoral and cell-mediated immune (CMI) responses, as they confer protection to homologous parental strains and partial protection to heterologous strains. Although it is possible to control some PRRSV outbreaks by use of MLV in combination with good practices, there are major safety issues such as a high mutation rate leading to reversion to virulence and recombination among vaccine and wild type strains. Cases have been reported in which new viruses have been introduced as a consequence of MLV vaccines. For example, nucleotide sequence identities of atypical Danish isolates were between 99.2 and 99.5% with the vaccine virus RespPRRS and 99.0–99.3% with VR2332, which is the parental virus to the vaccine virus, supporting the conclusion that the introduction of PRRSV-2 in Denmark was due to the spread of vaccine virus (109). In China a recombination event was reported in which a PRRSV variant with nucleotide deletions and insertions in the non-structural protein 2 (nsp2) gene also showed a possible recombination event between a MLV strain and a prototype Chinese field strain (110).

Current inactivated vaccine approaches are not highly effective since elicited immune responses are not enough to prevent spreading of the virus. However, this type of vaccine can augment anamnestic virus neutralizing antibodies and virus-specific IFN-γ responses following a wild-type virus infection or PRRSV-MLV vaccination which can contribute to viral clearance (111, 112). Thus, the combination of modified live vaccines with inactivated ones can be a reasonable approach to control the disease under field conditions (113) but unfortunately, there is no robust data comparing this approach with other options available on the market. On the other hand, most inactivated vaccines are not approved for use in the United States due to the poor efficacy showed in challenge trials (114) as measured by production of PRRSV specific neutralizing and non-neutralizing antibodies and low cellular immune responses leading to their failure in the porcine market. According to the Centre for Food Security and Public Health of Iowa State University, only “BIOSUIS PRRS Inact EU+Am” is approved to be used in the US. However, new strategies are being evaluated to overcome these problems (115), including nanoparticle entrapped antigens (116–119), plant based approaches (120) or vectored vaccines (121).

Several attempts have been made to use structural proteins to develop vaccines against PRRSV because they are specific targets of neutralizing antibodies. For this reason, one may hypothesize that antibodies against those proteins could be the main key to inhibit viral replication and spread as it is common for many viruses. Approaches such as VLPs combining different structural proteins have been tested (122–124), finding that anamnestic response is possible (boosted IgG and IFN-γ producing cells) in previously vaccinated or infected pigs but not in the pre-challenge period. These structural proteins are able to prime the immune system, but no reduction of viremia was observed after challenge (123). Those results suggest that other viral proteins may be targeted to induce a protective response in pigs. A plausible explanation for this finding may be based on the presence of few neutralizing epitopes in their sequences, most of which are located in variable regions of the proteins, to the phenomena of glycan shielding for epitopes and to the high variability observed between PRRSV virus strains. Again, a critical gap of knowledge for PRRSV is to precisely characterize common epitopes that are present in all PRRSV strains. Epitopes responsible for generating an efficient immune response eliciting cross-protective immunity remained elusive. Taken together, this evidence points to the need for new vaccination approaches that comply with a pathogen free strategy, capable of eliciting effective cellular and antibody responses with mid to long term protection against homologous strains and preferable to heterologous challenge as well.

### Extracellular Vesicles As a New Vaccination Approach

Extracellular vesicles(EVs) are gaining increased scientific attention as novel vaccines against infectious diseases, including animal diseases of veterinary importance by its capacity of self-antigen presentation, activation of host cell and antibody immune responses and more important, to induce protection in lethal challenge trials (125–131) (Box 2). In the case of PRRSV, artificial microRNAs (amiRNA) were initially synthetized to try suppressing expression of sialoadhesin (Sn) or CD163 by recombinant adenoviral vectors to be contained in exosomes, causing a subexpression of Sn and CD163 at mRNA and protein level, and reducing viral titter when porcine macrophages were pre-treated with amiRNA thus providing new evidence supporting the hypothesis that EVs can also serve as an efficient small RNA transfer vehicle for pig cells (132). More recently, PRRSV viral proteins associated to extracellular vesicles (EVs) in the size range of exosomes, were reported (129). Moreover, a targeted-pig trial using EVs from sera of infected pigs who had overcome the disease, demonstrated that EVs are capable of inducing specific IFN-γ secreting cells after a prime-boost strategy, are safe, free-of-virus and can differentiate infected from vaccinated animals (133), moreover, it was demonstrated that those EVs contained antigenic viral proteins recognized by pig immune sera and not by the pre-immune one. Of interest, however, a recent article indicated that PRRSV derived EVs are capable of transmitting the virus from one cell to another (134). Whether these discrepancies are due to *in vivo* vs. *in vitro* experimental work and methods applied to isolate EVs from serum samples or culture supernatant, remains to be determined.

Box 2 **Exosomes and therapeutic applications in PRRSV.**

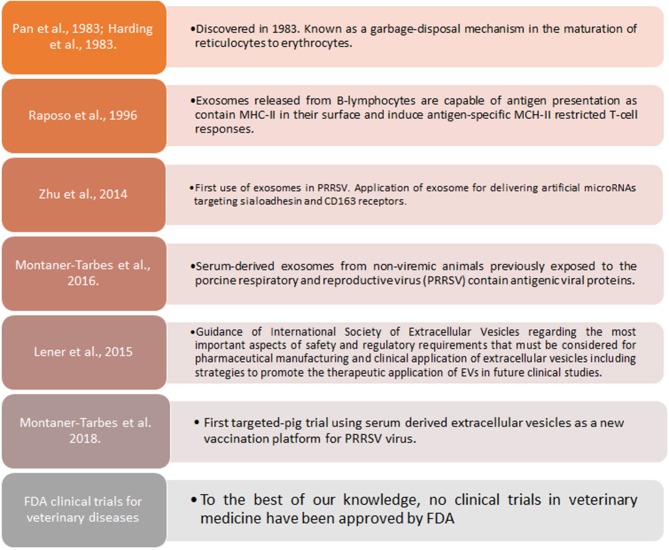

EVs have also been explored as novel control strategies in other viral diseases. For example, in respiratory syncytial virus infection, EVs are released with a selected modified cargo when compared with uninfected epithelial cells. When analyzed in detail, several viral proteins and diverse species of RNA were detected and capable of activating innate immune responses through induction of cytokine and chemokine release (135). Similar scenarios of viral proteins exported in EVs have been observed and extensively reviewed for HIV/HCV/HTLV-1 (136), EBV (137), and other viral diseases. Moreover, viral products of various origin and size including Ebola Virus VP24, VP40, and NP, Influenza Virus NP, Crimean-Congo Haemorrhagic Fever NP, West Nile Virus NS3, and Hepatitis C Virus NS3, when fused with Nef C-terminal domain through DNA vectors, were directed to the EVs membrane or packaged into them and remained stable after fusion. More importantly, when injected in mice, DNA vectors expressing the diverse fusion products elicited a well detectable antigen- specific CD8+ T cell response associating with a cytotoxic activity potent enough to kill peptide-loaded and/or antigen-expressing syngeneic cells, proving its promising results as a cytotoxic T lymphocyte vaccine (138).

### Concluding Remarks

PRRSV is a complex disease and several gaps in the knowledge of its economic impact, biology and evolution, genetic polymorphism, mechanism of viral infections, elicitation of protective immune responses and novel control strategies, have been reviewed here (Box 1). Since the late 1980's, different approaches have permitted to examine more closely this virus allowing the discovery of new features of the complex replication cycle, the identification of proteins and nucleic acids playing a role together with extracellular vesicles and nanotubules in facilitating spreading, and a better understanding of immune evasion (non-neutralizing antibodies, glycan shielding, mutation, recombination events, among others) to further vaccine development. Presently available PRRSV vaccines have many limitations in terms of heterologous protection, but some efforts have been made by combining new adjuvant formulations with modified live viruses, DNA and peptide vaccines, as well as extracellular vesicles a new vaccination approach. Advancing in all these gaps in knowledge, will eventually accelerate eliminating and eventually eradicating this devastating veterinary disease of such huge economic importance.

## Author Contributions

SM-T: wrote the first draft of the manuscript. MM, HdP, and LF: wrote sections of the manuscript. All authors contributed to manuscript revision, read, and approved the submitted version.

### Conflict of Interest Statement

The authors declare that the research was conducted in the absence of any commercial or financial relationships that could be construed as a potential conflict of interest. The reviewer SG declared a past supervisory role with one of the authors MM to the handling editor.
